# Family Communication at the End of Life

**DOI:** 10.3390/bs7030045

**Published:** 2017-07-14

**Authors:** Maureen P. Keeley

**Affiliations:** Department of Communication Studies, Texas State University, 601 University Drive, San Marcos, TX 78666, USA; Maureen.keeley@txstate.edu; Tel.: +1-512-245-3133

**Keywords:** communication, family, end of life, death and dying, palliative care, healthcare

## Abstract

People often feel awkward and ill at ease when faced with the opportunity for communication at the end of life, thus the overall theme for the articles in this special issue is the creation of more awareness and knowledge regarding the depth, breadth, and importance of current research exploring family communication at the end of life. This introductory essay attempts to accomplish the following: (1) discuss the importance of talk regarding death; (2) highlight the formative role of family interactions on the death and dying process; and (3) outline the articles in this special issue. Scholars contributing to this special issue on “Family Communication at the End of Life” have provided evidence that communication is important between and for terminally ill individuals, family members, and healthcare/palliative care specialists. Overall, research exploring communication at the end of life is especially relevant because every person experiences the death and loss of loved ones, and ultimately faces the reality of their own death.

The articles that compose this special issue focus on communication, families, and the end of life. Why is family communication at the end of life important? Taking a communication viewpoint on death and dying highlights the messages (both verbal and nonverbal) that occur between individuals pertaining to the topic and/or circumstances of death and dying [1]. Scholarly and clinical attention to communication at the end of life is imperative to improve medical, psychological, and relational outcomes for those dealing with the death and dying process (i.e., individuals who are terminally ill, as well as their close family members). However, communication at the end of life can be wrought with challenges, as many societies possess a belief of avoidance regarding death and dying [2]. Many reasons for avoidance regarding death and dying have been suggested by scholars such as: fear [3], cultural norms [4], religious beliefs [5], and/or family’s views of death as a taboo topic [6,7]. In the American Culture, where the majority of people die in hospitals, death has been routinely denied, sterilized, and/or removed from view [8]. Talking about dying is not morbid, nor is it magical talk that invites death into peoples’ lives, as some cultures believe [9], but it is often uncomfortable for family members [10]. It is uncomfortable because communication is a skill that takes practice and, in general, people have no experience with communication at the end of life; these interactions usually occur in private and behind closed doors [1]. It is not surprising that individuals feel awkward and ill at ease when faced with the opportunity for communication at the end of life. Thus, the overall theme for the articles in this special issue is the creation of more awareness and knowledge regarding the depth, breadth, and importance of current research exploring family communication at the end of life. This introductory essay attempts to accomplish the following: (1) discuss the importance of talk regarding death; (2) highlight the formative role of family interactions in the death and dying process; and (3) outline the articles in this special issue.

Communication at the end of life, before there is an impending death, can help remove the stigma that surrounds the topic of death and dying [1]. Talking about dying with the person that is terminally ill can relieve anxiety for both participants in the conversation and it can help ensure that final wishes regarding treatment at the end of life are honored [11]. Final conversations between the terminally ill and their family members can help individuals begin the grieving process while their terminally ill loved one is still present and can help in the process; it can help family members move on after the death without regret because nothing was left unsaid; and it can help individuals grow from the experience [10].

Focusing on the family at the end of life brings to the forefront the importance of family members and their interactions with the terminally ill for many reasons. Family members are very often the primary caregivers at the end of life and spend the most time with the terminally ill loved one [1]. Individuals’ daily routines and interactions are the most impacted throughout the end of life journey [12]. Family members are the ones that deal with the outcomes following the death of their loved one, therefore they have the most investment in the communication that occurs at the end of life [7]. In addition, family members are essential at the end of life because of the role they play as decision-makers and their responsibility in fulfilling the terminally ill’s final wishes [13]. Lastly, family members are the primary communicators with healthcare and palliative care professionals (e.g., doctors, nurses, clergy, social workers, et al.) regarding the care and health decisions surrounding the end of life journey of their dying loved ones [14].

The dying process for the terminally ill or aging is not a journey taken alone; it is a journey that is taken in the company of family members and loved ones, healthcare workers (e.g., doctors, nurses, clergy, etc.), and if fortunate enough, palliative care specialists. When the terminally ill have the opportunity and the openness to freely talk about what is on their minds and hearts, the end result is often relief of stress, more peaceful interactions, and greater readiness for the impending outcome [8]. How does effective communication at the end of life accomplish these positive outcomes? This is best highlighted through four explanations. First, the desires of the terminally ill become a priority and their voice is heard more clearly [15]. Second, both the family members and the terminally ill are more open, accepting, and ready for the end of life journey, therefore the terminally ill may be admitted earlier rather than later into Hospice. Patients that enter Hospice often have a better-quality end of life, with less pain and suffering, as well as a network of important social support and direction for families [15]. Third, the mutual acceptance of the impending death decreases the use of futile medical involvement near death that prolongs the inevitable and often leads to more pain for the terminally ill and more anguish for the family members as they watch their loved one suffer [15]. Fourth, participants are more willing to have the more intimate and authentic conversations that really should occur between family members and their dying loved ones at the end of life, creating a sense of closure and completion of their relationships [10].

With the goal of understanding the role, impact, and importance of communication at the end of life, researchers have been conducting research in earnest for the past 20 years focusing on both the communication between the terminally ill and health/palliative care professionals [7,16], and the communication between the terminally ill and their family members [1,16]. Communication scholars have been building a foundation of information and creating a body of knowledge pertaining to the importance of effective family communication at the end of life [17].

The fifteen articles that are included in this special issue on “Family Communication at the End of Life” are written by experts that focus primarily on communication at the end of life. Some of the authors have been working in this area for 20 years, while others are new scholars who represent the future directions for investigating communication at the end of life. All have a passion to help the everyday person struggling with the impending loss of a loved one in their family and to help the healthcare and/or palliative care professionals that work to improve their communication with the terminally ill, their family members, and their health coworkers. This special issue includes both quantitative (numbers that enable researchers to make generalizations) and qualitative methods (descriptions of people’s experiences) of inquiry highlighting the importance of a variety of methods for examining communication at the end of life. The diversity of methodology underscores the importance of different questions and perspectives on the investigation of family communication at the end of life.

Upon reflection, there are five major themes in this special issue exploring family communication at the end of life. The first area focuses on the new trend for communities and individuals that want to take the mystery and fear out of death and dying through communication. Specifically, the authors examine the importance of and the approaches for beginning the conversation about death and dying earlier rather than later. Amongst the three articles, one creates the argument that the communication about end of life should begin far earlier than is the current norm in their article “Upstreaming and Normalizing Advance Care Planning Conversations—A Public Health Approach” [18]. The second and third articles discuss unique situations to begin the conversations of death and dying. In the articles “Death Cafés: Death Doulas and Family Communication” [19] and “Contradictions and Promise for End-of-Life Communication among Family and Friends: Death over Dinner Conversations” [20], the authors highlight and analyze new ways to begin these conversations about death and dying. Talking about death and dying in these safe and voluntary environments, rather than in the midst of a terminal illness situation, may help to alleviate fear of death and normalize communication at the end of life.

The second theme of this special issue focuses upon who is making decisions and how they are made at the end of life. One article, “Designing Effective Interactions for Concordance around End-of-Life Care Decisions: Lessons from Hospice Admission Nurses” [21], highlights the insight gained from Hospice professionals regarding the important role that family members can and should play with their terminally ill loved one in the decision-making process regarding end of life care. The second article, “Family Communication about End-of-Life Decisions and the Enactment of the Decision-Maker Role” [22], focuses upon who in the family becomes the decision-maker for the family at the end of life and the facets that impact that role adoption, as well as the challenges that the chosen person faces in making the final decisions for the terminally ill.

The third theme highlights how age and diseases that come with getting older requires changes in how families communicate at the end of life. With the growing population in this era of “Baby Boomers” these articles begin a much needed discussion for families. One article, “How Older Adults and Their Families Perceive Family Talk about Aging-Related EOL Issues: A Dialectical Analysis” [23], focuses on the tensions inherent in communication at the end of life when it is complicated by aging issues. The second article, “Dementia at the End of Life and Family Partners: A Symbolic Interactionist Perspective on Communication” [24], suggests that family members can learn to live with their loved one at the end of life in new ways that can be fulfilling and satisfying for all parties involved. This article also acknowledges the challenges that are inherent when faced with a loved one with dementia at the end of life and offers suggestions for successful ways to communicate with them.

The fourth theme includes four articles that underscore the importance of good (i.e., satisfying for participants, effective for addressing needs, fulfilling goals) communication between the terminally ill, family members, and health/palliative care professionals. All participants involved must become a cohesive team focused on managing a number of relevant issues at the end of life. For example, one article, “Cancer Communication and Family Caregiver Quality of Life” [25], emphasizes the importance of acknowledging the overwhelming stress on the family caregiver. The crux of this article acknowledges the stress and demands put upon the family caregiver(s) and suggests the importance of effective communication for improving the overall welfare of family members and by doing so it improves the circumstances for the terminally ill as well. A second article, “Communication Matters: Exploring the Intersection of Family and Practitioner End of Life Communication” [26], acknowledges the inherent tension between the family’s desire for good care versus their acceptance that a cure is no longer a realistic goal, as well as the role that healthcare providers play in helping family members manage this tension. This article provides pragmatic communication solutions and suggestions to facilitate useful and mindful end of life communication between and among family members and healthcare providers. A third article on this topic, “Physicians’ Religious Topic Avoidance during Clinical Interactions” [27], indicates that many doctors are ill-prepared to talk about religious/spiritual issues when talking with terminally ill patients and their family members. Religious/spiritual issues are very important to a majority of people at the end of life [4], therefore the avoidance of these conversations due to the discomfort, inexperience, or lack of knowledge on this topic by doctors impacts the overall quality and satisfaction of the interaction. A fourth article titled “Palliative Care and the Family Caregiver: Trading Mutual Pretense (Empathy) for a Sustained Gaze (Compassion)” [28] assesses the impact of family members’ health literacy and its influence on communication at the end of life, as well as the determination of what kind and degree of healthcare is provided at the end of life.

The fifth and final theme of this special issue brings to the forefront the importance of exploring, acknowledging, and valuing the perspective of the family members’ experiences and recollections of their communication at the end of life. One article, “Still Searching: A Meta-Synthesis of a Good Death from the Bereaved Family Member Perspective” [29], reviews a large body of research regarding the meaning of a good death. The authors conclude that family members may experience either a “good death” or a “bad death” of a their loved one based on a number of factors: issues pertaining to the final care experiences of their loved ones, perceptions of the interactions between themselves and their loved one, and their interactions with the palliative care health systems used during and following the death. The authors also provide suggestions on how to improve the quality of these experiences. A second article, “Communicatively Constructing the Bright and Dark Sides of Hope: Family Caregivers’ Experiences during End of Life Cancer Care” [30], highlights the fact that family members often focus on the tension between the hope for a “cure” (also known as “the bright side,” even if it is based on avoidance and/or fake perceptions) and the hope for a “good death” (also known as “the dark side,” acknowledging and accepting the impending death). The article highlights the role of communication for promising social support, prioritizing family, and managing the honesty of the situation). A third article, “Death of an Ex-Spouse: Lessons in Family Communication about Disenfranchised Grief” [31], focuses on the grief that is not acknowledged, is frequently disqualified, and often cannot be acknowledged publically because of social circumstances. The communication at the end of life and following the death in these circumstances is different but still important for the individual experiencing the loss and grief. A fourth article focusing specifically on the family perspective of the communication at the end of life journey, entitled “Final Conversations: Overview and Practical Implications for Patients, Families, and Healthcare Workers” [32], reviews twelve years of published research exploring personal communication from the family members’ viewpoint. Family members ultimately must go on living following the death, and in that process they recall and reflect on their final conversations and interactions experienced during the end of life journey for months and even years [1]. Therefore, communication at the end of life potentially has the greatest and longest-lasting impact on family members. The article briefly highlights the most common themes of final conversations, provides pragmatic suggestions regarding communication at the end of life for the terminally ill, family members—including children and adolescents [33,34], and palliative/healthcare professionals.

In conclusion, the scholars contributing to this special issue on “Family Communication at the End of Life” have provided evidence that communication is important for terminally ill individuals, family members, and healthcare/palliative care specialists. This research exploring communication at the end of life is especially relevant because every person experiences the death and loss of loved ones and ultimately faces the reality of their own death [10]. When the terminally ill and their loved ones (most often their biological, legal, or chosen family members) have the opportunity and the openness to freely talk about what is on their minds and hearts at the end of life, the end result is often the relief of stress, peaceful interactions, and greater readiness for the impending outcome [9]. Still, such talks are not without their challenges [3]. In addition, communication at the end of life between the terminally ill and family members results in more satisfying care and an increased sense of well-being at the end of life for the dying [7]. Ultimately, the communication that occurs at the end of life between the terminally ill, family members, and healthcare specialists are critical for a “good death,” because it is only through communication where peoples’ true wishes are heard, understood, and followed that their loved ones are left without regret [10]. True regret comes from what is not communicated at the end of life.

## References

[1] Keeley M.P. (2007). “Turning toward death together”: The functions of messages during final conversations in close relationships. J. Soc. Pers. Relatsh..

[2] Bullock K. (2011). The influence on culture on end-of-life decision making. J. Soc. Work End-Life Palliat. Care.

[3] Keeley M.P., Generous M. (2015). The Challenges of Final Conversations: Dialectical Tensions during End-of-Life Family Communication from Survivors Retrospective Accounts. South. Commun. J..

[4] Keeley M.P. (2004). Final conversations: Survivors’ memorable messages concerning religious faith and spirituality. Health Commun..

[5] Generous M., Keeley M.P. (2016). Topics avoided and neglected with terminally ill loved ones at the end of life. Death Stud..

[6] Ko E., Roh S., Higgins D. (2013). Do older Korean immigrants engage in end-of-life communication?. Educ. Gerontol..

[7] Ragan S., Wittenberg Lyles E., Goldsmith J., Sanchez-Reilly S. (2008). Communication as Comfort: Multiple Voices in Palliative Care.

[8] Kubler-Ross E. (1969). On Death and Dying.

[9] Kubler-Ross E. (1997). Living with Death and Dying.

[10] Keeley M.P., Yingling J.M. (2007). Final Conversations: Helping the Living and the Dying Talk to Each Other.

[11] Generous M., Keeley M.P. (2014). Creating and validating the final conversations (FCs) scale: A measure of end-of-life relational communication with terminally ill loved ones. J. Soc. Work End-Life Palliat. Care.

[12] Keeley M.P., Baldwin P. (2012). Final conversations phase II: Children and everyday communication. J. Loss Trauma.

[13] Carr D., Khodyakov D. (2007). Health Care Proxies: Whom Do Young Adults Choose and Why?. J. Health Soc. Behav..

[14] Goldsmith J., Wittenberg-Lyles E., Ragan S., Nussbaum J.F., Thompson T.L., Parrott R., Nussbaum J.F. (2011). Lifespan and end-of-life health communication. The Routledge Handbook of Health Communication.

[15] Bernacki R.E., Block S.D. (2014). American College of Physicians High Value Care Task Force. Communication about serious illness care goals: A review and synthesis of best practices. JAMA Int. Med..

[16] Wittenberg-Lyles E., Goldsmith J., Sanchez-Reilly S., Ragan S.L. (2010). Dying with Comfort: Family Illness Narratives and Early Palliative Care.

[17] Keeley M.P. (2016). Invited: Family Communication at the End of Life. J. Fam. Commun..

[18] Prince-Paul M., DiFranco E. (2017). Upstreaming and Normalizing Advance Care Planning Conversations—A Public Health Approach. Behav. Sci..

[19] Baldwin P.K. (2017). Death Cafés: Death Doulas and Family Communication. Behav. Sci..

[20] Lambert South A., Elton J. (2017). Contradictions and Promise for End-of-Life Communication among Family and Friends: Death over Dinner Conversations. Behav. Sci..

[21] Candrian C., Tate C., Broadfoot K., Tsantes A., Matlock D., Kutner J. (2017). Designing Effective Interactions for Concordance around End-of-Life Care Decisions: Lessons from Hospice Admission Nurses. Behav. Sci..

[22] Trees A.R., Ohs J.E., Murray M.C. (2017). Family Communication about End-of-Life Decisions and the Enactment of the Decision-Maker Role. Behav. Sci..

[23] Egbert N., Child J.T., Lin M.-C., Savery C., Bosley T. (2017). How Older Adults and Their Families Perceive Family Talk about Aging-Related EOL Issues: A Dialectical Analysis. Behav. Sci..

[24] Johnson C., Kelch J., Johnson R. (2017). Dementia at the End of Life and Family Partners: A Symbolic Interactionist Perspective on Communication. Behav. Sci..

[25] Wittenberg E., Borneman T., Koczywas M., Del Ferraro C., Ferrell B. (2017). Cancer Communication and Family Caregiver Quality of Life. Behav. Sci..

[26] Omilion-Hodges L.M., Swords N.M. (2017). Communication Matters: Exploring the Intersection of Family and Practitioner End of Life Communication. Behav. Sci..

[27] Villagran M.M., MacArthur B.L., Lee L.E., Ledford C.J.W., Canzona M.R. (2017). Physicians’ Religious Topic Avoidance during Clinical Interactions. Behav. Sci..

[28] Goldsmith J., Ragan S.L. (2017). Palliative Care and the Family Caregiver: Trading Mutual Pretense (Empathy) for a Sustained Gaze (Compassion). Behav. Sci..

[29] Tenzek K.E., Depner R. (2017). Still Searching: A Meta-Synthesis of a Good Death from the Bereaved Family Member Perspective. Behav. Sci..

[30] Koenig Kellas J., Castle K.M., Johnson A., Cohen M.Z. (2017). Communicatively Constructing the Bright and Dark Sides of Hope: Family Caregivers’ Experiences during End of Life Cancer Care. Behav. Sci..

[31] Tullis J.A. (2017). Death of an Ex-Spouse: Lessons in Family Communication about Disenfranchised Grief. Behav. Sci..

[32] Keeley M.P., Generous M.A. (2017). Final Conversations: Overview and Practical Implications for Patients, Families, and Healthcare Workers. Behav. Sci..

[33] Keeley M.P., Generous M., Baldwin P. (2014). Final conversations phase II: Children’s final conversation messages with dying family members. J. Fam. Commun..

[34] Keeley M.P., Generous M. (2014). Advice from children and adolescents on final conversations with dying loved ones. Death Stud..

